# Influence of functional orthodontic therapy on body posture and postural control in children and adolescents with Class II malocclusion

**DOI:** 10.1186/s12903-025-07574-y

**Published:** 2025-12-30

**Authors:** Candelaria Sommer, Fabian Holzgreve, David A. Groneberg, Christina Erbe, Daniela Ohlendorf

**Affiliations:** 1 Institute of Occupational, Social and Environmental Medicine, Goethe University Frankfurt, Frankfurt am Main, 60596 Germany; 2 Department of Orthodontics, University Medical Center of the Johannes Gutenberg-University, Medical Centre of the Johannes Gutenberg-University Mainz, Mainz, 55131 Germany

**Keywords:** Functional orthodontic treatment, Angle class II malocclusion, Mandibular function, Postural control, Upper body posture

## Abstract

**Background:**

Malocclusions in children and adolescents are not only associated with aesthetic and functional issues but may also influence body posture due to neurophysiological links between the temporomandibular system and the spinal column. This study aimed to evaluate how functional orthodontic treatment may affect mandibular function, upper body posture, and postural control in children with skeletal Angle Class II malocclusion, by comparing findings before and after treatment.

**Methods:**

Thirty-nine children (18 f/ 21 m; median age 11.1 years, IQR 9.46–12.00 years) with skeletal Class II malocclusion were treated with a Balters bionator. Cephalometric data, occlusal parameters (overjet, overbite, transversal widths), axiographic measurements, as well as three-dimensional analyses of spinal and postural parameters were recorded both before and after therapy.

**Results:**

Pre-post-skeletal changes: Orthodontic treatment led to significant skeletal improvements, including increased SNB° (*p* ≤ 0.001; 76.6° → 77.5°), reduced ANB° (*p* ≤ 0.001; 4.5° → 2.9°), and decreased overjet (6.0 mm → 4.0 mm). Pre-post-mandibular changes: Mandibular movement remained symmetrical, while mouth-opening deviations dropped significantly from 92 to 15% (*p* ≤ 0.001). Pre-post-postural control, pre-post-upper body posture: No clinically relevant changes were observed in postural control or upper body posture—e.g., increased trunk length (D: + 18.18 mm, S: + 22.63 mm; both *p* ≤ 0.001). Balance distribution remained physiological (forefoot-rearfoot approx. 37:63%), sway parameters stayed within normal limits Correlations: Significant correlations emerged between mandibular mobility (especially left laterotrusion) and various postural and balance parameters, with low to moderate effect sizes. No associations were found between model analysis and either axiographic or postural measures.

**Discussion:**

This study underscores the functional stability of postural regulation in healthy children undergoing orthodontic treatment. Although significant improvements in dental and mandibular parameters were observed, neither postural control nor upper body posture exhibited clinically relevant changes. It should be noted that both posture and balance were already within physiological norms at baseline. In the absence of pre-existing postural dysfunctions, orthodontic interventions are unlikely to induce substantial postural changes.

## Introduction

Early orthodontic intervention in children and adolescents with malocclusions not only serves to prevent dental trauma, enhance oral hygiene by improving tooth alignment and achieve aesthetic benefits, but may also exert a positive influence on posture via neurophysiological connections between the temporomandibular system and the spinal column [1–7].

As early as 1928, Schwarz [8] postulated that a hyperkyphotic posture – particularly in the thoracic spine – could favour the development of mandibular retrusion. Bench [9] also highlighted the influence of cervical lordosis on craniofacial morphology, suggesting that individuals with pronounced cervical lordosis may exhibit a more square-shaped facial structure. The results of Gadotti et al. [10] indicated that participants aged 17 to 27 with Angle Class II malocclusion exhibited a significantly greater head–neck angle (mean: 40.4°) and a more pronounced anterior head posture compared to individuals with Angle Class I occlusion (mean: 36.4°), as measured from standardized lateral-view photographs taken in a standing position. In addition, Nobili et al. [11] examined 50 young adults with different malocclusions using a balance platform in different mandibular positions and showed a forward-shifted posture in Class II malocclusions, while Class III subjects demonstrated a backward-shifted posture. Lippold et al. [12] demonstrated two correlation patterns between the craniofacial morphology and the profile of the back shape in adults using videorasterstereography. One pattern showed a more distal and vertical craniofacial shape, with larger angles in the upper thoracic, lumbar and pelvic regions, while the other pattern showed a more mesial and horizontal craniofacial shape, with smaller angles in the upper thoracic, lumbar lordotic and pelvic regions.

Similar results are also available for children and adolescents. A comprehensive systematic review by Różańska-Perlińska et al. [13] showed a clear association between malocclusions and posture in children aged 12 to 15 years, while the evidence for a correlation with head posture and gait parameters was only moderate. According to Gresham and Smithells [14] certain findings related to the cervical spine appear to be associated with a skeletal distal bite position. Radiographic examinations in children revealed that those with a non-upright habitual head posture not only exhibited Angle Class II malocclusion, but also presented long face syndrome and an increased cervical lordosis**.** Gonçalves et al. [15] analyzed a cohort of children following the completion of orthodontic treatment for an anterior open bite or a posterior crossbite. An electromyographic evaluation of stomatognathic function was conducted seven days post-retention. The findings indicated an increase in the activity of the masseter and temporalis muscles during various static and dynamic mandibular functions and suggest that the correction of the malocclusion may induce a transient influence on the neuromuscular activity of the masticatory musculature.. These results indicate that orthodontic interventions not only modify dental occlusion, but may also induce functional adaptations within the neuromuscular system of mastication. In an interdisciplinary study of preschool children, a statistically significant association between scoliosis (*p* ≤ 0.03) and hypotonic posture (*p* ≤ 0.02) with Angle Class II malocclusion was demonstrated using video raster sterography [16]. The orthopaedic examination revealed that Angle Class II patients exhibited an accumulation of orthopaedic parameters, including scoliotic misalignment, hypotonic posture, fallen arches and pelvic obliquity.

Moreover, these studies underscore the importance of viewing orthodontic treatments in an interdisciplinary context. Given the documented interdependencies between occlusion, temporomandibular joint function, and spinal posture in children and adolescents, it is both reasonable and clinically relevant to examine these factors more closely in the context of orthodontic therapy. Recent research also highlights the relevance of functional occlusal disturbances for postural dynamics. Piancino et al. [17] demonstrated that posterior unilateral functional crossbite is associated with asymmetric spinal flexion, reinforcing the neuromuscular link between chewing patterns, functional orthodontic conditions, and both static and dynamic components of posture.

Earlier studies [18–20] have already shown that a functional orthodontic appliance in children causes changes in the temporomandibular joint and body posture, particularly in the head and neck region. Mertensmeier et al. [21] conducted a longitudinal study involving 126 children (Ø 10.6 years) over an average treatment period of 4.7 years with removable appliances. Compared to an untreated control group, both the Class I and Class II malocclusion groups exhibited a significant straightening of the cervical spine with mean changes of 3.4° and 2.6°, respectively. Klostermann et al. [20] investigated the relationship between posture and Angle Class II in children before and after early orthodontic treatment with removable, functional orthodontic appliances. Employing video raster stereography for postural assessment, the authors reported a significant reduction in overjet (mean: –3.9 mm ± 2.1 mm; *p* ≤ 0.05*),* which was accompanied by an improvement in pelvic torsion. There was also a significant, moderately pronounced correlation between the overjet change and pelvic torsion (*r* = 0.34, *p* ≤ 0.05).

Since the aforementioned studies primarily focused on dental and skeletal parameters, analyses related to the temporomandibular joint were largely omitted. Additionally, body posture and postural control were examined only in relation to occlusal characteristics. The present study aimed to provide a more comprehensive assessment by investigating the correlation between all relevant functional and structural parameters. These parameters included occlusion, temporomandibular joint function, upper body posture and postural control in children, both before and after functional orthodontic treatment (bionator according to Balters [22–26]was chosen as functional orthodontic appliance). Given that changes in occlusion and temporomandibular joint function in children and adolescents are often closely linked to developmental stages and growth dynamics [27], the potential impact of functional orthodontic treatment on upper body posture, mandibular mobility, and postural control may hold considerable clinical relevance. Therefore, this study investigated the effects and associations of functional orthodontic treatment with the Balters bionator [22–26] on skeletal parameters, occlusion, mandibular function (i.e., mandibular mobility and movement patterns assessed via jaw motion analysis), upper body posture, and postural control in children with skeletal Class II malocclusion before and after treatment. Based on previous findings and the physiological baseline posture of the participants, the following hypotheses were formulated:

1. Functional orthodontic treatment leads to significant skeletal improvements, including increased SNB°, decreased ANB°, and a reduction of overjet.
2. Functional orthodontic treatment does not induce clinically relevant changes in mandibular mobility or mandibular movement patterns.
3. Functional orthodontic treatment does not induce clinically relevant changes in upper body posture or postural control, as these parameters are expected to remain table within physiological norms.

## Materials and methods

All measurements were carried out in a private orthodontic practice by the same orthodontist. Each diagnostic procedure was performed at two standardized time points: (1) at baseline, immediately before initiation of functional orthodontic treatment, and (2) at follow-up, directly after completion of the therapy. Both measurement sessions included the full diagnostic protocol (cephalometric radiograph, model analysis, axiography, posturography, and spine scan).

### Subjects

A total of 39 children and adolescents (18 female, 21 male) aged between seven and 13 years were included in the prospective experimental study. The median age of the female participants was 11.0 years (1st quartile: 9.80/3rd quartile: 11.90), and the median age of the male participants was 10.8 years (1st quartile: 9.50/3rd quartile: 12.30).

The total median age at the beginning of the study was 11.1 years (1st quartile: 9.46/3rd quartile:12.00 years), whereas the median age after completion of functional orthodontic treatment was 12.10 years (1st quartile: 1050/3rd quartile: 13.05 years).

As part of the routine initial diagnostic assessment, all lateral cephalometric radiographs were additionally evaluated according to the cervical vertebral maturation method described by Hassel and Farman [28]. Although the anatomical evaluation is based on the morphology of vertebrae C2–C4, the resulting maturation stages are conventionally denoted with the prefix ‘S. All children were classified within skeletal maturation stages S1–S3, corresponding to a pre-pubertal developmental phase. In addition, the posterior airway space (PAS) was recorded as part of the standard cephalometric analysis, where its automated measurement provides a screening indicator of upper airway dimensions. Consistent with the skeletal Class II pattern of the sample, the majority of participants (67%) showed a narrowed PAS, whereas 33% presented with PAS values within the normal range.

All subjects were patients of a private orthodontic practice and were treated by one orthodontist. Before treatment they all completed a medical history form [29], which contained questions on general illnesses such as diabetes mellitus, tinnitus, osteoporosis and rheumatism. Information was also collected on joint pain, musculoskeletal complaints, headaches and migraines, temporomandibular joint noises, physician diagnosed pathologies of the musculoskeletal system, accidents and operations on the musculoskeletal system and medication intake. Children with previously diagnosed orthopaedic disorders (e.g. scoliosis, structural leg length discrepancy, congenital spinal deformities) were excluded based on a standardized parental questionnaire. In addition, a brief assessment of the temporo-mandibular system was carried out on all test subjects using the preventive manual structural analysis according to Bumann [30, 31].

In addition, routine orthodontic intake examinations were performed for all children, which included assessment of oral habits, tongue posture, swallowing pattern, and breathing mode as observed during clinical inspection, as well as general intraoral soft-tissue function. No standardized quantitative tests (e.g., validated myofunctional protocols, rhinomanometry, or ophthalmologic screening) were carried out, as these are not part of standard diagnostic procedures in private orthodontic practice in Germany. A systematic assessment of visual or oculomotor abnormalities was also not conducted, because no child presented any clinical signs of visual dysfunction during the initial anamnesis, and such assessments would have required specialized interdisciplinary testing (ophthalmology or orthoptics), which was not feasible within the design of this study.

The inclusion criteria were skeletal Class II malocclusion diagnosed on lateral cephalometric analysis, ageed between 7 and 13 years, no prior orthodontic treatment and good general health without musculoskeletal or neurological disorders. Skeletal Class II was identified using standard cephalometric parameters (ANB°, SNA°, SNB°). Dental characteristics typically associated with Class II (such as increased overjet and Class II molar relationship) were also documented; however, inclusion was strictly based on the cephalometric skeletal measurements.

Exclusion criteria included: syndromic or craniofacial anomalies, temporomandibular disorders requiring separate treatment, clinical signs typically associated with temporomandibular disorders such as joint noises, pain on palpation, or noticeable deviations during mandibular movement,systemic diseases affecting growth or posture, and incomplete pre- or post-treatment records. Additionally, if acute complaints or injuries to the musculoskeletal system, the use of muscle relaxants, medically diagnosed physical malpositions, ongoing physiotherapy or orthopaedic therapies, structural orthopaedic diseases, general illnesses with long-term therapy, syndromes, physical or mental disabilities were identified (recorded using a medical history form [29]), they were excluded from the study.

The subjects were instructed to wear the functional orthodontic appliance for 14 h per day.

Since functional treatment was indicated based on the individual skeletal Class II malocclusion, all patients were treated with the same appliance (bionator according to Balters [22–26]) to ensure methodological consistency. Treatment goals included improvement of the sagittal jaw relationship and reduction of overjet.

Sample size calculation is as follows: with a sample size of 39 patients, correlations of strength 0.46 (i.e., moderate effects according to Evans) or greater can be demonstrated significantly (alpha = 5%, beta = 20%).

Before the study began, the parents or guardians of all participants under the age of 16 have given their consent for participation. All radiographic examinations were performed strictly on medical indication and in compliance with the European Council Directive 2013/59/Euratom and the ALARA principle [32]. In accordance with German legal requirements, parental consent was obtained verbally as part of routine clinical diagnostics, with all documentation maintained by the treating orthodontic practice.

This study was conducted in accordance with the local Ethics Committee of the Department of Medicine at Goethe University (No. 103/16) and the principles of the 1964 Declaration of Helsinki and its subsequent amendments.

### Measurement methods and evaluation parameters

#### Cephalometric radiograph

Lateral cephalometric radiographs were obtained digitally and evaluated using the Ivoris® software system (Computer konkret GmbH, Germany). Standardized cephalometric landmarks and reference planes were identified digitally. All measurements were recorded before and after treatment to assess skeletal and dental changes associated with functional orthodontic therapy. Lateral cephalometric radiographs were taken at two time points: at baseline prior to functional orthodontic treatment and at follow-up after completion of therapy.

All radiographic procedures were performed in accordance with the “As Low As Reasonably Achievable” (ALARA) principle, following German radiation protection regulations and clinical orthodontic standards [32].

##### Calibration

The cephalometric radiographs were obtained using a certified radiographic system that undergoes mandatory quality assurance according to the German Radiation Protection Act/Radiation Protection Ordinance.

This includes annual constancy and quality control checks performed by authorized service engineers as well as routine daily system checks before clinical use.

Because radiographic systems operate with fixed and legally certified calibration standards, no additional individual calibration is required before each exposure.

#### Model analysis

The orthodontic model analysis was carried out completely digitally. An intraoral scanner (iTero Element™ 2, Align Technology Switzerland GmbH) was used to create three-dimensional intraoral scan of teeth in the upper and lower jaw. The captured scan data were digitally evaluated for further analysis. The transversal dental arch width of both jaws, the overjet and overbite, sagittal space conditions, midline shift in the upper and lower jaw and the occlusal conditions of the first molars on the right- and left side, including the angle classifications, were recorded. Model analysis was carried out at two time points: before initiation of functional orthodontic treatment and after completion of the treatment period.

#### Axiography

**Fig. 1:**
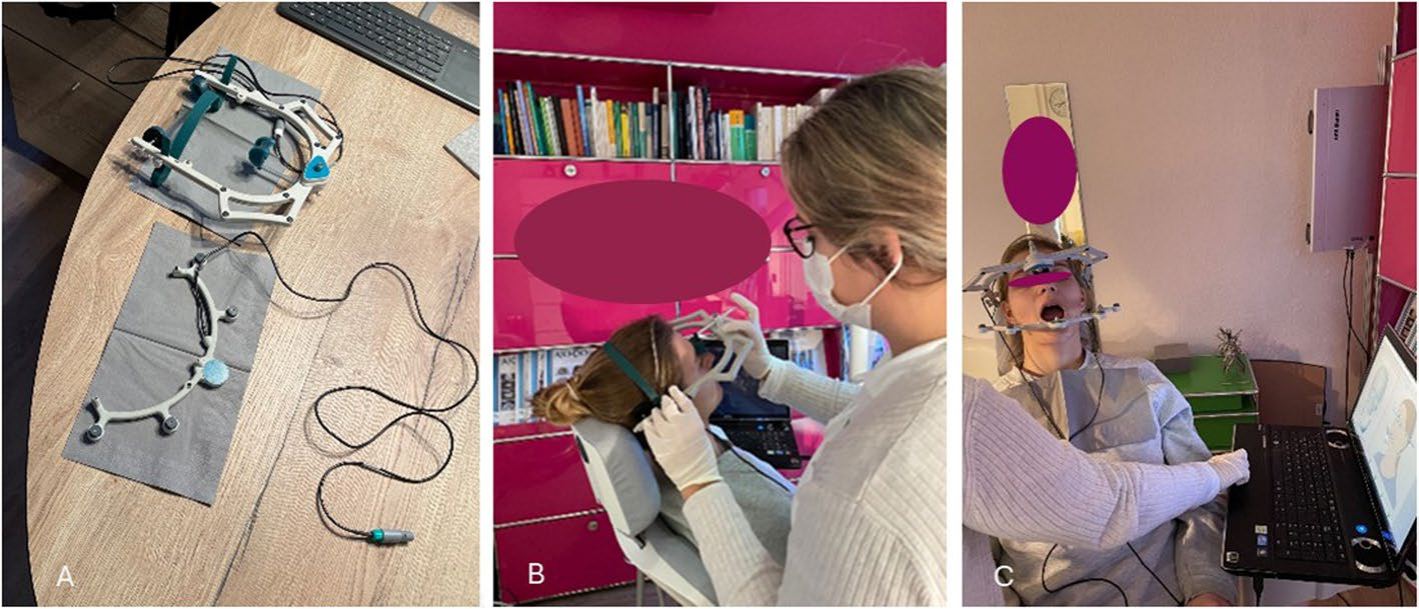
**a**-**c** Depiction of the individual axiography devices divided into a face bow for the upper jaw in the upper image and a registration tray for the lower jaw in the bottom part of the image in 1 A, placement and adjustment of the upper face-bow and headgear to prepare for mandibular motion registration in 1B and dynamic recording of the mandibular opening movement in1C.

The Jaw Motion Analyzer system (Zebris Medical GmbH, Isny) was used for the functional analysis of the temporomandibular joint. This system is based on an ultrasound-based method that uses a measuring sensor to record the mobility of the mandible with a frequency of 50 Hz and an accuracy of 0.1–0.2 mm [33, 34]. The jaw movements were registered using a paraocclusal tray that was fixed with bite registration material (“Luxabite”, DMG). The data was analysed using the WinJAW + software (version 1.0) (Fig. 1a-c).

The Zebris JMA system requires a patient-specific calibration procedure before each examination. This step is part of the standardized measurement protocol and includes sensor alignment, mandibular reference calibration and functional verification of mandibular movements. The calibration was performed immediately before each test session to ensure measurement accuracy and reproducibility.

**Fig. 2:**
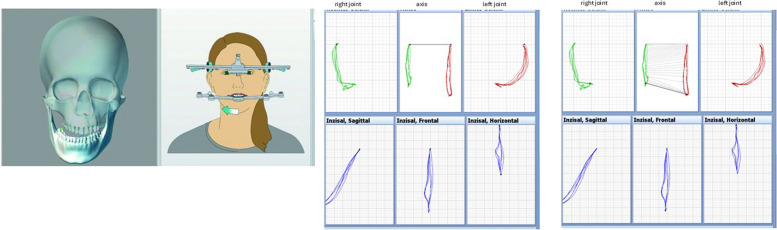
Left: 3D skull model with visualized mandibular left sided laterotrusion. Right: Graphical analysis of condylar and incisal point movements for mouth opening. The upper row displays right and left condylar trajectories in sagittal, frontal, and horizontal projections. The lower row shows the corresponding incisal point paths for mouth opening

The measurement protocol comprised three consecutive cycles, which were averaged to record laterotrusion, protrusion, mouth opening, deviation, and deflection (Fig. 2).

Laterotrusion was divided into three groups: normal (7–12 mm), hypofunctional (< 7 mm) and hyperfunctional (> 12 mm) [35]. The accepted, normal protrusion values were set at 7–11 mm and for the mouth opening at 40–60 mm [35]. Normality thresholds for laterotrusion, protrusion and maximum mouth opening were defined according to established functional diagnostic norms [36] and pediatric reference values [37]. Lateral deviations in mouth opening were recorded using the frontal Posselt diagram and assessed with regard to symmetry (deviation vs. deflection). Functional axiography was performed at baseline and at the post-treatment follow-up to evaluate changes in mandibular movement patterns.

#### Three-dimensional back scanner

**Fig. 3:**
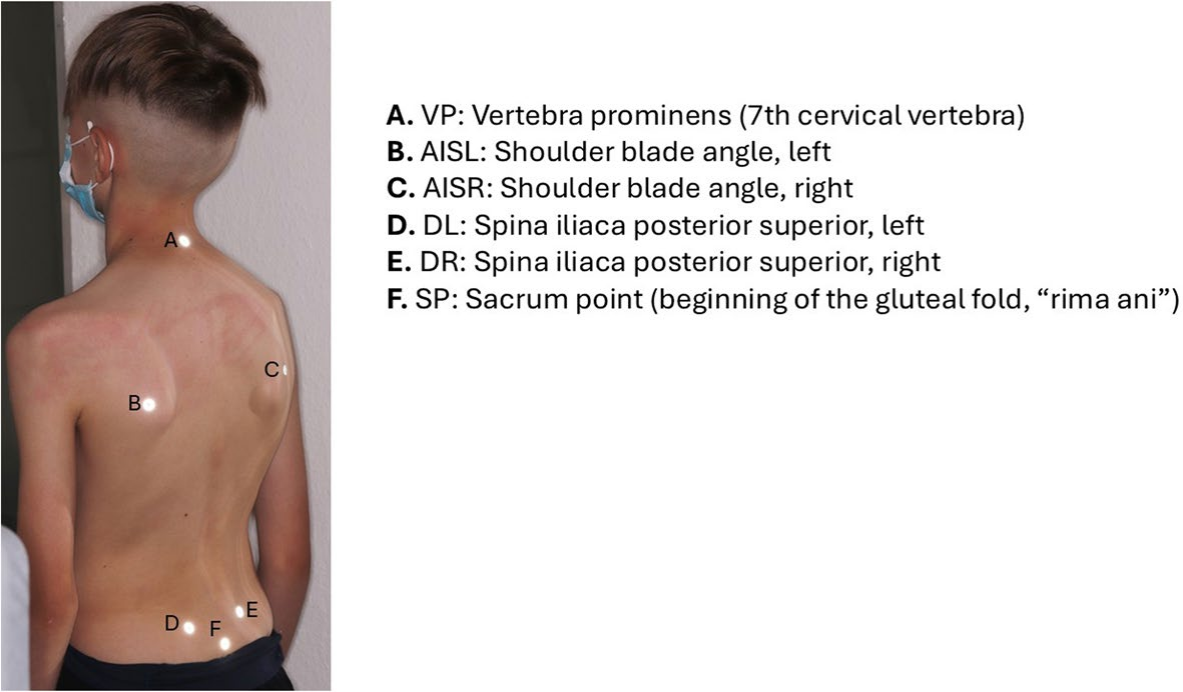
Six anatomical landmarks on the bare back for capturing upper body posture

The “ABW Mapper mobile” (ABW GmbH, Frickenhausen/Germany) is used to measure the back in three dimensions without contact or radiation. For a precise image of the back surface, 30 video images are recorded per second, with a maximum frame rate of 50 images per second. The resulting image is presented in a three-dimensional representation with a spatial depth resolution of 1/100 mm. The manufacturer states that the measurement error is less than 1 mm. Repeated measurements show a reproducibility of less than 0.5 mm. A projector is integrated into this device that projects a striped pattern onto the surface of the spine. An LCD camera with a resolution of 640 × 480 pixels and a size of 600 × 400 mm records the pattern from a defined angle to enable a triangulation technique for further evaluation. In this study, to assess the dorsal upper body posture, six anatomical fixed points on the bare back were marked with reflective markers for the spine, shoulder and pelvic regions and calculated from two markers each (Fig. 3). This procedure can be used to assess changes in the shoulder and pelvic area (both sagittal and frontal changes) as well as the shape of the spine (lordotic, kyphotic or scoliotic deformities).

The ABW BackMapper system is factory-calibrated by the manufacturer at regular annual intervals. Because rasterstereographic systems rely on fixed geometric calibration parameters, no further manual calibration before individual measurements is required.

Several studies have demonstrated a high level of agreement between rasterstereographic surface topography and conventional radiographic imaging in the assessment of spinal posture: In particular, Drerup and Hierholzer [38] showed that rasterstereographic reconstructions of the spinal midline exhibit low root-mean-square deviations in both lateral deviation and vertebral rotation when compared with radiographic gold standards. These findings have been supported by subsequent systematic reviews and meta-analyses, which reported consistently high validity and reliability of rasterstereography for quantifying spinal posture and curvature parameters [39, 40].

Spinal posture measurements were obtained at baseline and at follow-up after functional orthodontic treatment.

#### Posturography

**Fig. 4:**
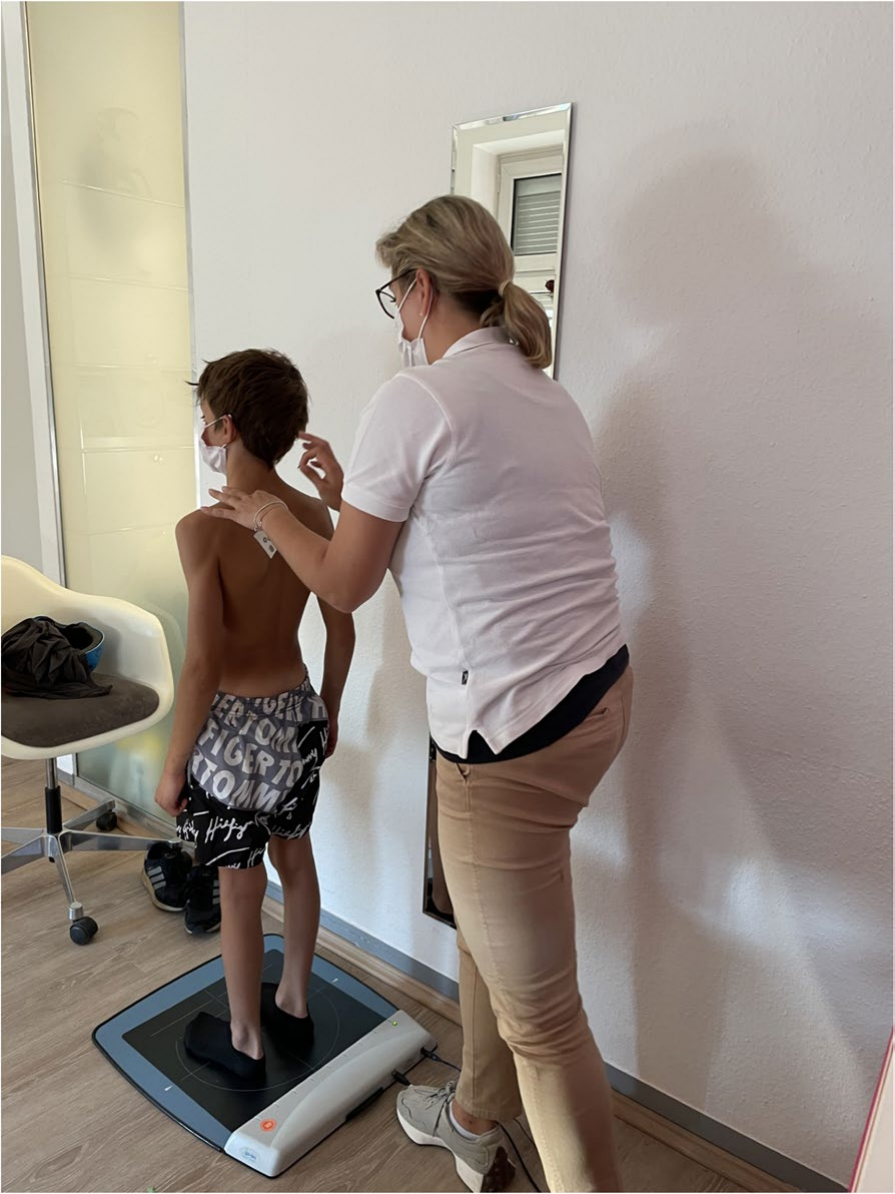
A patient standing on the pressure measurement plate with the marker for the back scan attached so that postural control and back scanning can take place simultaneously

Posturography was performed using the GP MultiSens pressure measuring plate (GeBioM GmbH, Münster, Germany). This sensor plate has a measuring surface of 38.5 cm × 38.5 cm and is equipped with 2,304 matrix-shaped sensors (Fig. 4). Each sensor has a size of approximately 8.8 mm. The measuring frequency is 100 Hz per sensor, resulting in a total sampling rate of approximately 500 kHz. The measurement error is ± 5%. Posturographic assessments were conducted at two time points: before functional treatment and after completion of the therapy.

The GP Multisens posturography platform undergoes manufacturer-performed annual factory calibration and routine maintenance. No additional user-dependent calibration is required, as the system operates with fixed certified calibration parameters provided by the manufacturer.

#### Evaluation parameter

Cephalometric radiograph: The following cephalometric variables were included in the analysis: Sella–Nasion–A-point angle (SNA°), Sella–Nasion–B-point angle (SNB°), A-point–Nasion–B-point angle (ANB°), overjet, overbite, and the inclination of the maxillary and mandibular incisors (U1-NA°, L1-NB°).

Model analysis: transversal dental arch width maxilla and mandible, horizontal and vertical anterior step, midline shift in the mandible, occlusion of the first molars on the right and left, and the angle classes.

Axiography: laterotrusion (left and right), protrusion, mouth opening, deviation, deflection (all parameters in mm).

Three-dimensional back scanner: Upper body posture is differentiated into the shoulder region, the spine region, and the pelvic region. The parameters for the three superordinate regions are listed and defined in Table 5.

Posturagraphy: Equilibrium fluctuation (frontal (FS) and sagittal (SS) sway in mm)), ellipse area (cm2), width (cm) and height (cm), balance (left–right (L/R) as well as the forefoot and rearfoot load (FF/RF) of the left and right foot (in %).

### Statistical evaluation methods

The statistical analysis was carried out using BIAS version 11.12 software (Epsilon Verlag, Darmstadt). To check for normal distribution, all data were tested using the Kolmogorov–Smirnov-Lilliefors test. For all parameters, based on their non-normal distribution, the median (including the 1 st and 3rd quartiles)**,** the confidence interval and the tolerance range were determined and non-parametric tests were used.

The Wilcoxon matched pairs test was used to test for differences. All p-values were subjected to a subsequent Bonferroni-Holm correction. The correlations were calculated using Kendall’s tau and Spearman’s rho with the correlation coefficient (*r*). The correlation coefficient was classified according to the following effect sizes according to Evans (1996): < 0.2 = low, 0.2–0.4 = weak, 0.4–0.6 = moderate, > 0.6 = strong. The significance level was set at *p* ≤ 0.05.

## Results

As numerous variables were collected as part of this study, only those results for which statistically significant changes were found in the before/after comparison and in the correlations are presented below. Therefore, the primary focus here was not on the completeness of all dental and skeletal parameters of the dynamic occlusion or axiography but rather on the relevance of the results shown in the tables. Thus, the focus here was on the before and after comparison.

### Orthodontic analysis

Table 1 describes the most relevant cephalometric angles in combination with the model analysis by the bionator therapy in children with an initial Class II malocclusion. There is a significant increase in the SNB° angle (*p* ≤ 0.001) and a reduction in the ANB° angle (*p* ≤ 0.001) with a moderate to strong effect (rho = 0.34 and 0.59, respectively). The overjet was significantly reduced from a median value of 6.00 mm before treatment to 4.00 mm after treatment (*p* ≤ 0.001; rho = 0.49) and represented a significant improvement in the sagittal anterior relationship. The values for the overbite, the SNA° angle and the inclination of the upper and lower incisors (maxillary NA° and mandibular NB°) did not change significantly.

**Table 1:** Changes in the orthodontic parameters before and after bionator therapy. Significant p-values after Bonferroni-Holm correction are represented in bold. Effect sizes are classified according to Evans: < 0.2 = low, 0.2–0.4 = weak, 0.4–0.6 = moderate, > 0.6 = strong

	**Before orthodontic treatment**				**After orthodontic treatment**					
Class II (*n* = 39)	**Median**	** 1 st quartile/3rd quartile**	**Confidence interval lower limit**	**Confidence interval upper limit**	**Median**	** 1 st quartile/3rd quartile**	**Confidence interval lower limit**	**Confidence interval upper limit**	***p*-value**	Correlation coefficient **rho**
SNA°	80.70	79.10/83.10	79.70	82.40	80.60	78.80/82.60	79.90	81.90	0.18	0.16
SNB°	76.60	75.20/78.40	75.90	78.00	77.50	76.00/79.50	76.90	78.80	**0.001**	**0.34**
ANB°	4.50	3.70/6.10	3.70	5.40	2.90	2.20/4.30	2.20	3.20	**0.001**	**0.59**
OK-NA °1	26.00	22.10/32.30	23.40	30.10	25.60	20.80/28.80	21.90	28.30	0.07	0.21
UK-NB°1	23.90	20.00/29.50	22.10	27.50	25.20	20.30/31.30	22.00	28.00	0.16	0.16
Overjet	6.00	4.00/7.00	4.50	6.50	4.0	2.30/5.0	3.50	4.00	**0.001**	**0.49**
Overbite	4.00	2.00/5.50	3.00	4.50	3.0	2.0/4.0	2.00	3.50	0.12	0.19

Table 2 shows changes in the model analysis and axiography before and after orthodontic treatment. The transversal width difference in the maxilla to the Berendok nominal value (mm) (*p* ≤ 0.001) increased significantly with a moderate effect size (rho = 0.45). The support zone difference on the right and left sides of the mandible compared to the target value according to Berendok (mm) decreased significantly (*p* ≤ 0.001), while left laterotrusion, protrusion and maximum mouth opening did not change significantly. In addition, a significant reduction in the overjet was observed; this decreased from a median value of 6.00 mm to 4.00 mm (*p* = 0.001; rho = 0.49). In contrast, the overbite showed no significant change (*p* = 0.10; rho = 0.21). In addition, there was a significant reduction in laterotrusion on the right (*p* = 0.01; rho = 0.31), while laterotrusion on the left, protrusion and maximum mouth opening did not change significantly.

**Table 2:** Changes in the orthodontic and functional parameters before and after bionator therapy in children with Class II malocclusion. Indications of the medians, 1 st and 3rd quartiles, lower and upper limit of confidence interval (CI), p-values and correlation coefficients (rho), including the classification of effect size according to Evans: < 0.2 = low, 0.2–0.4 = weak, 0.4–0.6 = moderate, > 0.6 = strong, are shown. Significant *p*-values after Bonferroni-Holm correction are represented in bold

		**Before orthodontic treatment**	**After orthodontic treatment**	*p*-value	Correlation coefficient rho
Upper jaw: Support zone difference to the right of the target value according to Berendok (mm)	Median	0.50	0.00	0.23	0.15
	1 st quartile	−0.80	−0.80		
	3rd quartile	1.30	1.20		
	CI lower limit	−0.70	−0.70		
	CI upper lmit	1.20	0.30		
Upper jaw: Support zone difference to the left of the target value according to Berendok (mm)	Median	0.30	0.00	0.44	0.10
	1 st quartile	−0.70	−0.80		
	3rd quartile	1.30	1.20		
	CI lower limit	−0,20	−0.70		
	CI upper lmit	1,00	0.30		
Upper jaw: Transversal width difference to target value according to Berendok (mm)	Median	−2.50	0.00	**0.001**	0.45
	1 st quartile	−4.00	−2.50		
	3rd quartile	0.00	1.00		
	CI lower limit	−3.00	−1.50		
	CI upper lmit	−1.00	1.00		
Lower jaw: Support zone difference to the right of the target value according to Berendok (mm)	Median	2.40	−0.20	**0.001**	0.44
	1 st quartile	0.40	−1.20		
	3rd quartile	3.70	2.00		
	CI lower limit	1.70	−0.60		
	CI upper lmit	3.40	1.40		
Lower jaw: Support zone difference to the left of the target value according to Berendok (mm)	Median	2.70	0.00	**0.001**	0.51
	1 st quartile	1.70	−0.50		
	3rd quartile	3.70	1.70		
	CI lower limit	2.00	−0.30		
	CI upper lmit	3.40	0.70		
Lower jaw: Transversal width difference to target value according to Berendok (mm)	Median	0.00	0.00	0.12	0.21
	1 st quartile	−1.00	−1.00		
	3rd quartile	3.00	0.00		
	CI lower limit	−0.50	−0.50		
	CI upper lmit	2.00	0.00		
Anterior stage Horizontal (mm)	Median	6.00	4.00	**0.001**	0.49
	1 st quartile	4.50	3.00		
	3rd quartile	7.00	5.00		
	CI lower limit	4.50	3.50		
	CI upper lmit	7.00	4.00		
Anterior stage Vertical (mm)	Median	4.00	3.00	0.10	0.21
	1 st quartile	2.00	2.00		
	3rd quartile	5.50	4.00		
	CI lower limit	3.00	2.00		
	CI upper lmit	5.00	7.50		
Laterotrusion right (mm)	Median	6.60	5.80	**0.007**	0.31
	1 st quartile	5.90	5.20		
	3rd quartile	7.80	6.90		
	CI lower limit	6.20	6.20		
	CI upper lmit	7.50	7.20		
Laterotrusion left (mm)	Median	6.50	6.00	0.20	0.15
	1 st quartile	5.00	4.90		
	3rd quartile	7.60	6.80		
	CI lower limit	5.70	5.70		
	CI upper lmit	7.20	1.10		
Difference left/right (mm)	Median	0.40	0.30	0.70	0.05
	1 st quartile	−1.20	−1.10		
	3rd quartile	1.40	1.20		
	CI lower limit	−0.80	−0.80		
	CI upper lmit	1.10	1.10		
Protrusion (mm)	Median	5.80	5.60	0.56	0.07
	1 st quartile	5.10	4.80		
	3rd quartile	6.50	6.10		
	CI lower limit	5.40	5.40		
	CI upper lmit	6.30	6.30		
Opening (mm)	Median	17.00	18.30	0.53	0.07
	1 st quartile	12.80	15.80		
	3rd quartile	22.80	19.80		
	CI lower limit	13.60	13.60		
	CI upper lmit	19.30	19.30		

Table 3 shows the mandibular deviation and deflection during mouth opening before and after orthodontic treatment. Before treatment, 7.69% (*n* = 3) of the subjects showed a deviation, with the deviation manifesting itself to the right in 5.13% (*n* = 2) of the cases and to the left in 2.56% (*n* = 1) of the cases. In addition, 92.31% (*n* = 36) of the children showed a mandibular deflection, which was directed to the right in 33.33% (*n* = 13) of the cases and to the left in 15.38% (*n* = 6) of the cases. After orthodontic treatment, the prevalence of deviation remained unchanged at 7.69% (*n* = 3) (5.13% (*n* = 2) right, 2.56% (*n* = 1) left). Mandibular deflection, on the other hand, decreased significantly and was only present in 15.38% (*n* = 6) of the subjects. Right deflection was observed in 12.82% (*n* = 5) of the subjects, while left deflection was only present in 2.56% (*n* = 1) of the cases.

**Table 3:** Deviation and deflection during mouth opening with the direction of deviation, distribution in percent (%) and number (n) before and after orthodontic treatment

	Available at	Deviation direction	
		Right	Left
Before orthodontic treatment			
Deviation	7.69% (*n* = 3)	5.13% (*n* = 2)	2.56% (*n* = 1)
Deflection	92.31% (*n* = 36)	33.33% (*n* = 13)	15.38% (*n* = 6)
After orthodontic treatment			
Deviation	7.69% (*n* = 3)	5.13% (*n* = 2)	2.56% (*n* = 1)
Deflection	15.38% (*n* = 6)	12.82% (*n* = 5)	2.56% (*n* = 1)

### Effects of orthodontic treatment on balance distribution, postural sway and upper body posture

Table 4 shows the results of the posturography before and after orthodontic treatment. After Bonferroni-Holm correction, no significance was observed. Descriptively, orthodontic treatment resulted in a slight shift from the forefoot to the hindfoot, with a balanced left–right distribution after orthodontic treatment. The proportion of left hindfoot loading increased from 28.00% to 35.15%, while right hindfoot loading decreased from 35.30% to 29.30%. Furthermore, the frontal deviation was reduced from 33.30 mm to 29.70 mm and the sagittal deviation increased from 24.00 to 27.00 mm. After orthodontic treatment, the ellipse area and ellipse width were minimally reduced from 5.00 cm² to 4.50 cm² and from 2.10 cm to 1.80 cm, respectively, and the ellipse height from 0.70 cm to 0.80 cm.

**Table 4:** Comparison of the parameters of postural control in the groups “before orthodontic therapy” and “after orthodontic therapy”. The medians, 1 st and 3rd quartiles of the posturographic parameters are shown. Significant p-values after Bonferroni-Holm correction are represented in bold. Effect sizes are classified according to Evans: < 0.2 = low, 0.2–0.4 = weak, 0.4–0.6 = moderate, > 0.6 = strong

	Before orthodontic treatment		After orthodontic treatment			
	Median	1 st quartile/3rd quartile	Median	1 st quartile/3rd quartile	p-value	Correlation coefficient rho
Forefoot left (%)	18.30	12.00/25.70	17.70	10.70/28.70	0.56	0.07
Forefoot right (%)	18.30	10.30/23.70	16.30	8.70/22.30	0.91	0.01
Rearfoot left (%)	28.00	21.30/35.30 35,15	35.15	24.30/43.30	0.18	0.15
Rearfoot right (%)	35.30	25.00/40.00	29.30	18.00/35.70	**0.01**	0.30
Left foot (%)	50.00	40.00/60.00	52.00	41.00/59.00	0.76	0.04
Right foot (%)	50.00	43.00/57.00	48.00	32.00/68.00	0.06	0.21
Forefoot (%)	37.00	29.30/45.00	32.30	26.30/44.30	0.27	0.13
Rearfoot (%)	63.00	55.00/71.70	67.70	53.30/71.70	0.42	0.10
Frontal sway (mm)	33.30	21.00/63.70	29.70	22.30/48.70	0.06	0.22
Sagittal sway (mm)	24.00	9.00/51.30	27.00	17.30/38.00	0.30	0.12
Ellipse area (cm^2^)	5.00	2.10/15.50	4.50	1.70/9.50	0.07	0.21
Ellipse width (cm)	2,10	1.40/3.40	1.80	1.40/2.80	0.08	0.20
Ellipse height (cm)	0,70	0.40/1.20	0.80	0.40/1.10	0.26	0.14

Table 5 compares the mean and median values of the back parameters before and after orthodontic treatment. The values for trunk length D (d = 18.18 mm) and trunk length S (d = 22.63 mm) were found to be statistically significant with a *p*-value of ≤ 0.000 and a large effect size (trunk length D: rho = 0.50; trunk length S: rho = 0.59). The measured values for sagittal trunk tilt had a difference of 5.04° and were also significant (*p* ≤ 0.02), although the effect size was low (rho = 0.25). The results for pelvic distance 2 also demonstrated statistical significance with a p-value of ≤ 0.03 and a clinical difference of 2.94°, however, here the effect size was also low at rho = 0.24. After Bonferroni-Holm correction, all *p*-values > 0.001 were no longer statistically significant, thus, the sagittal trunk tilt and pelvic distance 2 were no longer statistically significant. All other parameters were significant in the comparison.

**Table 5:** Comparison of the back parameters in the groups “before orthodontic therapy” and “after orthodontic therapy”. The medians, 1 st and 3rd quartiles of the back parameters are shown; significant p-values after Bonferroni-Holm correction are represented in bold. Effect sizes are classified according to Evans: < 0.2 = low, 0.2–0.4 = weak, 0.4–0.6 = moderate, > 0.6 = strong

Spine parameter	Before orthodontic treatment		After orthodontic treatment			
	Median	1 st quartile/3rd quartile CI lower limit CI upper lmit	Median	1 st quartile/3rd quartile CI lower limit CI upper lmit	p-value	rho
Shoulder region						
Scapula angle left (°) Angle of the compensation line applied from the shoulders to the horizontal. The centre of the compensation line is specified vertically above AISL	27.15	22.23/ 33.57 24.81 31.07	25.65	20.37/ 30.71 22.64 29.70	0.12	0.18
Scapula angle right (°) Angle of the compensation line applied from the shoulders to the horizontal. The centre of the compensation line is specified vertically above AISR	28.89	23.25/ 35.40 24.11 33.08	29.32	23.32/ 33.57 25.56 32.88	0.88	0.02
Scapula distance (mm) Distance between the left (AISL) and the lower right scapular angle (AISR)	174.44	158.40/ 204.67 366.05 397.84	181.30	166.70/ 196.94 379.38 420.39	0.43	0.09
Scapula height (mm) Height difference between the AISL and AISR points	−2.21	−6.72/ 0.33 381.65 425.10	−2.23	−6.57/ −0.58 414.53 449.97	0.91	0.01
Scapula rotation (°) Rotation of the distance AISL—AISR in the transversal plane	2.82	0.28/ 5.45 −3.48 −1.05	2.42	−0.61/ 4.22 −4.66 −1.96	0.77	0.03
Spine region						
Trunk length D (mm) Spatial distance between the markers C7 and middle of the PSIS marker	383.31	353.41/ 403.56 0.32 1.62	401.49	372.80/ 435.31 0.23 1.36	**0.001**	0.50
Trunk length S (mm) Spatial distance between the markers at C7 and the rima ani	409.88	375.11/ 430.32 −0.64 1.15	432.51	397.90/ 462.63 −1.16 0.39	**0.001**	0.59
Sagittal trunk decline (°) Inclination of the trunk length D marked line from the perpendicular to the sagittal plane	−1.96	−3.88/ 0.09 14.45 17.10	−3.08	−4.93/ −1.03 14.00 16.46	0.02	0.25
Frontal trunk decline (°) Inclination of the trunk length D marked line from the perpendicular to the frontal plane	0.79	0.04/ 1.72 161.04 195.31	0.90	−0.08/ 1.66 170.26 193.65	0.76	0.04
Axis decline (°) Deviation of the line of the area marked by the trunk length D line of the 90° rotated distance between PSIS left and PSIS right	0.20	−1.35/ 1.71 −4.69 0.28	−0.49	−1.93/ 0.82 −5.24 −1.31	0.06	0.22
Thoracic bending angle (°) Deviation of the distance C7 – Kyphosis apex from the perpendicular	15.60	13.18/ 18.28 0.49 4.74	16.02	12.97/ 16.83 1.33 4.13	0.40	0.09
Standard deviation of lateral deviation (mm) Root mean squared deviation of the median line of the distance C7 – centre of the PSIS marker	3.55	2.77/ 5.30 3.22 5.52	3.48	2.63/ 4.47 2.96 4.18	0.65	0.05
Standard deviation of rotation (°) Root mean squared deviation of surface rotation of the median line (torsion of the spinous processes of the spine)	5.39	2.65/ 7.13 3.09 6.44	3.81	2.70/ 5.82 2.82 5.04	0.07	0.20
Kyphosis angle (°) Angle between the upper turning point at C7 and the thoracolumbar inflection point	46.30	38.07/ 54.02 40.44 53.05	44.75	38.35/ 50.78 40.37 49.01	0.65	0.05
Lordosis angle (°) Angle between the lower inflection point at the centre of the PSIS marker and the thoracolumbar turning point	39.53	27.98/ 44.48 33.97 42.87	34.10	28.12/ 40.16 29.43 37.72	0.13	0.18
Lumbar bending angle (°) Deviation of the distance Kyphosis apex – Lordosis apex from the perpendicular	12.07	8.68/ 13.81 77.55 89.89	11.37	8.75/ 14.98 80.08 93.77	0.16	0.16
Pelvis region						
Pelvic distance (mm) Spatial distance between the left (PSISL) and right (PSISR) of the pelvis	84.45	76.53/ 92.63 −1.35 0.04	83.85	78.47/ 95.89 −1.72 −0.58	0.41	0.10
Pelvic obliquity angle (°) Decline of the connecting line between PSIS left and PSIS right to the horizontal in the frontal plane in degrees	−0.80	−1.52/ 0.59 −2.22 0.04	−1.32	−2.03/ −0.34 −2.44 −0.92	0.02	0.23
Pelvic obliquity (mm) Decline of the connecting line between PSIS left and PSIS right to the horizontal in the frontal plane in millimetres	−1.18	−2.50/ 0.88 −2.35 2.71	−1.76	−2.88/ −0.51 −3.63 0.93	0.03	0.24
Pelvis torsion (°) PSIS left – PSIS right, twist around the transverse axis calculated from the mutual twisting of the surface normal on the two PSIS	0.24	−3.11/ 3.61 0.03 3.50	−1.45	−4.42/ 1.29 −0.59 2.32	0.06	0.21
Pelvis rotation (°) Rotation of the distance PSIS left – PSIS right in the transversal plane	1.82	−0.82/ 3.87 9.91 13.24	0.62	−1.91/ 3.92 10.05 13.03	0.86	0.02

### Correlations between the axiography or model analysis and the upper body posture, as well as the postural control before and after orthodontic therapy

#### Correlations between axiography and the upper body posture or postural control

This section relates the data of the axiography, with the maximum movement limits of the mandible, and the occurrence of deviations of the mandible from the centre at mouth opening, in relation to the parameters of the back. Table 6 summarizes the correlations found between laterotrusion to the right or left and the parameters of the back or the postural control parameters, both before and after treatment. There were no correlations found between laterotrusion to the right with postural control or the upper body posture at either measurement time.

**Table 6:** Correlations between the dynamic occlusions and upper body posture. The p-values and effect sizes (rho) for correlations between the axiography and the back parameters of postural control are shown before and after orthodontic treatment. Effect sizes are classified according to Evans (1996): < 0.2 = low, 0.2–0.4 = weak, 0.4–0.6 = moderate, > 0.6 = strong

	**Lat R before orthodontic treatment**		**Lat L before orthodontic treatment**		**Lat R after orthodontic treatment**		**Lat L after orthodontic treatment**		**Effect size**
	***p*-value**	**rho**	***p*-value**	**rho**	***p*-value**	**rho**	***p*-value**	**rho**	
Upper body posture									
Scapula angle left (°)	0.09	0.276	0.66	0.07	0.76	0.05	0.67	0.07	low
Scapula angle right (°)	0.23	0.20	0.70	0.63	0.60	0.09	0.77	−0.05	low
Trunk length D (mm)	0.91	−0.02	0.82	0.04	0.08	0.28	0.65	−0.07	low
Trunk length S (mm)	0.64	−0.08	0.70	0.06	0.12	0.25	0.78	−0.05	low
Sagittal trunk decline (°)	0.59	0.09	0.27	0.18	0.34	−0.14	0.03	−0.35	weak
Frontal trunk decline (°)	0.80	−0.04	0.16	0.22	0.90	−0.02	0.05	0.32	weak
Axis decline (°)	0.60	0.85	0.35	0.15	0.65	0.07	0.002	0.49	moderate
Thoracic bending angle (°)	0.84	−0.03	0.12	0.25	0.43	0.13	0.74	−0.05	moderate
Scapula distance (mm)	0.40	−0.14	0.26	0.18	0.17	0.22	0.65	0.07	low
Scapula height (mm)	0.71	−0.06	0.03	−0.34	0.55	0.10	0.33	0.16	low
Scapula rotation (°)	0.84	−0.03	0.94	−0.01	0.87	0.03	0.34	0.16	low
Standard deviation of lateral deviation (mm)	0.63	−0.08	0.60	0.09	0.28	−0.18	0.21	−0.21	weak
Standard deviation of rotation (°)	0.24	−0.19	0.76	0.05	0.89	0.03	0.77	0.05	low
Kyphosis angle (°)	0.82	−0.04	0.66	0.07	0.71	0.06	0.18	−0.22	weak
Lordosis angle (°)	0.53	−0.10	0.90	−0.02	0.66	−0.07	0.20	−0.21	weak
Pelvic distance	0.58	−0.10	0.72	−0.06	0.05	0.31	0.45	0.12	low
Pelvic height (°)	0.88	0.02	0.85	−0.03	0.45	0.12	0.07	0.29	weak
Pelvic height (mm)	0.75	0.05	0.99	0.00	0.71	0.06	0.06	0.23	weak
Pelvis torsion (°)	0.93	−0.01	0.85	−0.03	0.62	−0.08	0.98	0.00	low
Pelvis rotation (°)	0.15	−0.23	0.09	−0.27	0.55	0.10	0.02	0.37	weak
Lumbar bending angle (°)	0.25	0.19	0.24	0.19	0.64	0.08	0.36	−0.15	low
Plantar pressure distribution									
Forefoot left (%)	0.98	0.00	0.56	−0.09	0.43	−0.13	0.34	0.16	low
Forefoot right (%)	0.75	0.05	0.84	0.03	0.51	0.12	0.02	−0.37	weak
Rearfoot left (%)	0.75	0.05	0.45	−0.12	0.52	−0.12	0.07	−0.29	weak
Rearfoot right (%)	0.77	−0.05	0.87	0.03	0.31	−0.17	0.33	0.16	low
Left foot (%)	0.96	−0.00	0.36	−0.15	0.89	−0.02	0.69	−0.07	low
Right foot (%)	0.99	0.00	0.33	0.16	0.89	0.02	0.69	0.07	low
Forefoot (%)	0.75	−0.05	0.72	0.06	0.81	−0.04	0.37	−0.15	low
Rearfoot ((%)	0.61	0.08	0.93	−0.01	0.81	0.04	0.37	0.15	low
Frontal sway (mm)	0.56	−0.09	0.30	−0.17	0.16	−0.23	0.02	−0.36	weak
Sagittal sway (mm)	0.96	−0.01	0.72	0.06	0.58	0.10	0.23	−0.20	weak
Ellipse area (cm^2^)	0.97	−0.01	0.98	−0.00	0.97	0.01	0.16	−0.23	weak
Ellipse width (cm)	0.89	0.02	0.64	−0.08	0.56	−0.09	0.02	−0.37	weak
Ellipse height (cm)	0.91	−0.02	0.71	0.06	0.87	0.03	0.31	−0.17	low

Before orthodontic therapy, laterotrusion to the left correlated with the scapula position (*p*-value = 0.03, rho = −0.34) with a moderate effect. Thus, a greater laterotrusion to the left was associated with a reduced scapula position. There were no significant correlations found between laterotrusion to the left and right with the postural control parameters (*p* ≥ 0.05).

After orthodontic therapy, laterotrusion to the right correlated positively and significantly with the pelvic distance (*p* = 0.05; rho = 0.31). The effect size was in the medium range at rho = 0.31. Laterotrusion to the left correlated positively and significantly with axial deviation (*p* = 0.002; rho = 0.49), frontal trunk tilt (*p* = 0.05; rho = 0.32) and pelvic rotation (*p* = 0.02; rho = 0.37), and significantly and negatively with sagittal trunk tilt (*p* = 0.03; rho = −0.35). A moderate effect size was observed for all significances.

There were no significant correlations between right laterotrusion and the postural control parameters (*p* ≥ 0.05). However, there was a significant negative correlation (*p*-value = 0.02, rho = −0.37) between the laterotrusion on the left and the forefoot on the right with a moderate effect size. There was also a significant correlation with a moderate effect size (p-value = 0.02, rho = −0.36) between the laterotrusion on the left and the frontal sway and a significant correlation between the laterotrusion on the left and the ellipse width (*p* = 0.02, rho = −0.37).

#### Correlation between the model analysis and postural control

Table 7 illustrates the correlation calculations between the horizontal anterior step and the posturographic parameters before and after orthodontic treatment. No correlations could be determined for any of the parameters in the model analysis (*p* ≥ 0.05).

**Table 7:** Correlations between the horizontal anterior step and the parameters of postural control. Effect sizes are classified according to Evans (1996): < 0.2 = low, 0.2–0.4 = weak, 0.4–0.6 = moderate, > 0.6 = strong

	**Horizontal anterior step before orthodontics**		**Horizontal anterior step after orthodontics**		**Effect size**
	***p*-value**	**rho**	***p*-value**	**rho**	
Forefoot left (%)	0.68	−0.07	0.66	−0.07	low
Forefoot right (%)	0.20	−0.21	0.48	−0.12	low
Rearfoot left (%)	0.86	−0.03	**0.02**	0.39	weak
Rearfoot right (%)	0.34	0.14	0.85	−0.03	low
Left foot (%)	0.92	0.02	0.33	0.16	low
Right foot (%)	0.99	−0.02	0.33	−0.16	low
Forefoot (%)	0.45	−0.12	0.15	−0.23	weak
Rearfoot ((%)	0.33	0.16	0.15	0.23	weak
Frontal sway (mm)	0.43	−0.13	0.22	−0.20	weak
Sagittal sway (mm)	0.34	0.14	0.97	−0.01	low
Ellipse area (cm^2^)	0.45	0.13	0.34	−0.56	moderate
Ellipse width (cm)	0.99	−0.00	0.10	−0.23	weak
Ellipse height (cm)	0.38	0.14	0.78	−0.05	low

#### Correlations between the model analysis and the axiography

No significant correlations were found between the model analysis and the axiography according to the following correlations:

- Support zone difference upper jaw right vs. protrusion/opening/laterotrusion right/laterotrusion left
- Support zone difference upper jaw left vs. protrusion/opening/laterotrusion right/laterotrusion left
- Transversal width difference upper jaw vs. laterotrusion left + right/mouth opening
- Support zone difference lower jaw right vs. protrusion/opening/laterotrusion right/laterotrusion left
- Support zone difference lower jaw left vs. protrusion/opening/laterotrusion right/laterotrusion left
- Transversal width difference lower jaw vs. laterotrusion left/laterotrusion right/protrusion/mouth opening
- Horizontal anterior step vs. protrusion/mouth opening
- Vertical anterior step vs. protrusion/mouth opening

#### Summary of statistically significant and clinically relevant findings

Table 8 summarises all statistically significant and clinically relevant results.

**Table 8:** Summary of statistically significant and clinically relevant findings

*Analysis*	*Parameter*	*p-value*	*Correlation* *Coefficient (rho)*	*Effect size* *Evans*
Cephalometric radiograph	ANB°	**0.001**	**0.59**	moderate
	Overjet	**0.001**	**0.49**	moderate
Model	Upper jaw transverse width difference to target value according to Berendok (mm)	**0.001**	**0.45**	moderate
	Lower jaw:Support zone difference to the right of the target value according to Berendok (mm)	**0.001**	**0.44**	moderate
	Lower jaw:Support zone difference to the left of the target value according to Berendok (mm)	**0.001**	**0.51**	moderate
	Anterior stage horizontal (mm)	**0.001**	**0.49**	moderate
Back scan	Trunk length D	**0.001**	**0.50**	moderate
	Trunk length S	**0.001**	**0.59**	moderate
Correlation: axopgraphy vs. back scan	Axis decline vs. -Lateralflexion Left after orthodontic treatment	**0.002**	**0.49**	moderate

## Discussion

The cephalometric analysis demonstrated the successful correction of malocclusions in children with skeletal Class II malocclusion by the Balters Bionator, particularly for the SNB° and ANB° angles, as well as a significant reduction in overjet. A comparison of the present data with established, population-specific longitudinal cephalometric data from Stahl de Castrillon et al. [41] on untreated German children with normal occlusion between the ages of 6 and 17 shows treatment-related changes in expected physiological growth changes. The post-treatment SNA, SNB, and ANB values fell within the age-appropriate reference ranges [41] and the observed shifts in the sagittal jaw relationship correspond to the direction typically expected from functional orthopedic treatment. These results are consistent with previous studies documenting positive skeletal changes occuring with functional orthodontic appliances [19, 20]. The first hypothesis can therefore be verified. The analysis of the mandibular movement showed that laterotrusion to the left, protrusion and mouth opening remained stable. Laterotrusion to the right decreased significantly (*p* = 0.01; rho = 0.31) although this was within the functional normal limits. Overall, movement coordination remained symmetrical. The second hypothesis can therefore be verified. The prevalence of deflections in mouth opening was 92.31% before treatment which decreased to 15.38% after the completion of treatment. The frequency of deviations remained constant at 7.69%. The successful occlusion correction had no negative effects on the upper body posture. Even before the treatment, the children showed a physiologically balanced posture with a greater kyphosis angle than lordosis angle (46.30° and 39.53°, respectively). The third hypothesis can therefore be verified. The documented increase in trunk length should be viewed in the context of natural length growth in childhood [13], while the minimal change in trunk tilt (1.12° more forward tilt), although statistically significant, is clinically unremarkable. Other parameters of upper body posture, such as pelvic rotation or axial deviation, also remained within the normal range. The study by Klostermann et al. [20] showed a significant reduction in the overjet (Ø −3.9 mm ± 2.1 mm; *p* ≤ 0.05) that was associated with an improvement in pelvic torsion and other postural parameters; the authors interpreted these changes as an indication of a positive influence of functional orthodontic measures on body posture. Compared to our results, however, these effects appear to be rather small and without clear clinical relevance. A conclusive evaluation remains elusive, thus, further long-term studies are necessary in order to classify in more detail the functional significance of such correlations. The children's posture remained largely unchanged over the entire treatment period.

Even at the beginning, physiologically stable posture was observed that did not change significantly as a result of the therapy, a finding that is consistent with previous studies [42, 43]. The balance shift was already harmonious before treatment – with a distribution of 50:50 left–right and 37:63 forefoot-rearfoot – and remained so over the course of the treatment. This observation corresponded to the normal body weight distribution for children and adults from other studies [44, 45]. The increased hindfoot loading was due to the centre of mass of the human body at the level of the promontory [46]. The postural control remained clinically unremarkable (frontal sway (33.30 mm → 29.70 mm); sagittal sway (24 mm → 27 mm)), as well as the slight reduction of the ellipse area (5.00 cm2 → 4.50 cm2). These values were above the reference values of Pomarino et al. [47] for 7–10-year old children (2.02 ± 0.43 cm2) but are should be evaluated within the age-related variance. The postural stability present in the age group studied is also supported by earlier studies [48, 49] that showed that although primary school children show greater fluctuations than adults, they become more stable with increasing age. Significant correlations between the mandibular mobility and body posture emphasise these functional interactions: greater laterotrusion to the left correlated with increased axial deviation to the left side (*p* ≤ 0.002; rho = 0.49), increased pelvic rotation (*p* ≤ 0.02; rho = 0.37) and lower trunk forward tilt (*p* = 0.03; rho = –0.35). The more pronounced the laterotrusion to the right, the greater is the distance between the pelvic markers (*p* ≤ 0.05; rho = 0.31). These results indicate that changes in mandibular mobility can result in adjustments in pelvic balance and the spinal alignment with low to moderate effect sizes. Correlations between the mandibular mobility and postural control revealed that increased left laterotrusion was associated with a reduction in right hindfoot load (*p* ≤ 0.02; rho = –0.37) and reduced frontal sway (FSW) with a moderate effect size (*p* ≤ 0.02; rho = –0.36). Such correlations indicate functional linkages, however, due to the low effect sizes, they are more likely to be indicative of trends rather than clinically relevant correlations. Overall, the orthodontic therapy had barely any functional influence on the children's posture. At the same time, it should be taken into account that, despite statistical inconspicuousness at the group level, individual differences in upper body posture may exist. Earlier studies [12, 19, 20] confirm the results observed in the present study; in children without initial postural deficits, no significant changes or compensations in the area of posture were observed in the course of orthodontic treatment. Whether this is due to a possible functional independence of the examined systems, or is due to compensatory processes cannot be conclusively assessed on the basis of the available data. Perinetti et al. [7] also found no evidence of postural changes caused by orthodontics in healthy children. The present cohort had already shown a physiologically stable posture at the start of treatment; neither the weight distribution nor the sway parameters deviated from the age-appropriate normal values. This stable starting position explains the absence of treatment-related postural changes, a finding that is supported by comparable studies [42, 50, 51]. Even with statistically significant correlations between the occlusal and postural parameters, the effect sizes were low to moderate, thus, clinically relevant compensation patterns can be ruled out. The functional significance of such correlations remains open and should be further investigated using longitudinal studies with differentiated group selection.

This study also had limitations. The study referred to a specific paediatric patient group with skeletal Angle Class II malocclusions, aged seven to 13 years. Age- and development-specific influences, as well as individual growth and neurophysiological maturation during the study period, may have influenced the results. Transferability to other age groups or occlusal classes is, therefore, only possible to a limited extent. We considered sex-specific developmental differences in craniofacial growth and postural control. However, cervical vertebral maturation analysis confirmed that all participants were assessed before the pubertal growth spurt, establishing a uniformly prepubertal or early pubertal cohort. While sexual dimorphism is well-documented, it typically becomes pronounced only after puberty. As our participants were predominantly in the prepubertal or early pubertal phase (7–13 years), sex-related distinctions in craniofacial structure and postural control were expected to be minimal, a finding supported by developmental research [52–54]. Studies on craniofacial and postural development, including the systematic review by Różańska-Perlińska et al. [13], highlight that functional and postural parameters in children exhibit substantial interindividual variability before mid-puberty, with stable, sex-dimorphic patterns typically emerging only later in adolescence. Similarly, normative data [55] indicate that age- and sex-related differences in mandibular movement capacity between 7 and 13 years are small relative to the overall variability in this age range. Given this developmental heterogeneity and the limited sample size, stratified analyses by age group and sex would have been statistically underpowered and potentially misleading. For this reason, and consistent with previous pediatric studies using comparable age ranges, we analyzed the cohort as a whole.. Nevertheless, as only healthy, symptom-free children were included, the risk of systematic bias was low. The absence of an untreated control group, a methodological limitation in distinguishing between treatment effects and physiological growth, can be addressed by descriptive comparison with age- and gender-specific longitudinal cephalometric reference data [39]. Another limitation is the absence of a no-treatment control group. Due to German radiation protection regulations and the ALARA/ALADA principles [32], additional radiographic exposure is ethically impermissible in children without a clinical indication. This restriction, which makes obtaining longitudinal material virtually impossible today [41], is compounded by epidemiological data (DMS VI [56]) showing that over half of German children undergo orthodontic treatment, precluding the recruitment of a large, untreated cohort. Comparable contemporary studies [18, 20] also omit radiographic control groups for the same ethical reasons. To contextualize our findings, we utilized the age- and sex-specific normative cephalometric values for untreated German children from Stahl de Castrillon et al. [41] as a reference. In addition, a limitation is the inability to perform a detailed assessment of the craniocervical and craniomandibular relationship (C1–C2) including potential rotational deviations. A reliable evaluation of atlas–axis position and asymmetry would require three-dimensional imaging (CBCT), as two-dimensional lateral cephalograms are insufficient due to superimposition. However, CBCT imaging cannot be ethically justified in an asymptomatic pediatric cohort without a specific diagnostic or therapeutic indication in accordance with current radiation protection standards and the ALARA/ALADA [32] principles. Consequently, a more comprehensive analysis of craniocervical interrelationships was not feasible within the design of this study. Furthermore, the evaluation of the posterior airway space on lateral cephalometric radiographs must be interpreted with caution. Measurements derived from two-dimensional images are susceptible to technical and functional variability, as the complex three-dimensional morphology of the upper airway cannot be fully represented on 2D projections. Transient soft-tissue movements (e.g., swallowing, tongue posture) at the moment of exposure can temporarily reduce the visible airway space, potentially leading to an underestimation of patency.

While three-dimensional or dynamic imaging (e.g., CBCT) provides more reliable volumetric information, such techniques are not feasible in routine pediatric diagnostics due to radiation exposure and ethical constraints (ALARA/ALADA[32]) [57, 58]. However, standardised orthopaedic or neurological initial examination was not carried out, thus, undetected compensation patterns could not be completely ruled out. Inter-examiner reliability was not applicable as all specialized measurements (functional axiography, posturography, and 3D spinal analysis) were performed exclusively by a single, highly experienced examiner. Intra-examiner variability was systematically minimized through the use of automated digital acquisition systems (Zebris JMA, GP MultiSens, ABW BackMapper), which inherently reduce subjective influence. Furthermore, each assessment was performed in triplicate and averaged, ensuring high precision and consistency without the need for a separate formal reliability study.

The measurements of postural control were performed in a standardised manner on a standing pressure plate (for 30 s, repeated three times and then averaging the results). Although longer measurement times (e.g. 90 s according to Ruhe et al. [59]) are recommended, other studies [60] show that shorter intervals also provide valid results when repeated several times. The jaw movement analysis using Zebris® required a high level of cooperation from the young test subjects.Additionally, the accuracy of the axiography system used in children is limited due to coordination challenges [61]. Younger children, in particular, had difficulties performing the Posselt diagram. And despite preliminary exercises, some subjects showed uncoordinated movement patterns, particularly when performing the mandibular movements required to trace Posselt’s envelope of motion; however, repeated efforts and standardized instructions aimed to reduce inconsistency. This may have affected the accuracy of the individual parameters (e.g. deviation, deflection). In addition, the measurement systems used have not been primarily validated for children and, thus, this entails technical limitations.

Although some of the results indicate relevant trends, no clear clinical conclusions can be drawn from them. However, the data obtained do provide a valuable basis for further research approaches. It would be desirable to improve the quality of the axiography for use with children and adolescents since performing the movements in this study was associated with coordination difficulties. A more child-friendly technical implementation or specific preliminary exercises could improve, significantly the measurement quality in the future. All subjects had a skeletal Angle Class II malocclusion and were treated exclusively with a Balters Bionator. In the future, it would seem sensible to examine homogeneous groups within the angle classes in a differentiated manner, particularly with regard to Class III patients. Furthermore, other functional orthodontic appliances should be examined in a similar way. As children with known orthopaedic pre-existing conditions and acute musculoskeletal complaints were excluded from the present study, it is not possible to assess whether orthodontic therapy would have had different effects in children with pre-existing postural weaknesses. Due to the largely normal physiological starting position of the subjects included, possible effects in children with pre-existing conditions cannot be assessed on the basis of these data.

## Conclusion

The aim of the present study was to investigate potential relationships between dental or dental skeletal parameters and postural control and upper body posture in healthy children aged between seven and 13 years during the course of orthodontic treatment. The children's posture was already largely physiological before the start of treatment, with respect to the axial body posture, almost symmetrical weight distribution (left–right 50:50, forefoot-rearfoot distribution in the range of 37:63) and postural fluctuation values that were within the age-appropriate normal ranges [13, 44]. No systematic changes in upper body posture were observed during the course of treatment. The few significant correlations between the mandibular mobility and posture showed moderate effect sizes at best and indicate functional interactions rather than clinically relevant adjustments. This largely physiological initial situation at the beginning of treatment could explain why no compensatory or structural adjustments were necessary and why the orthodontic intervention can be classified as functionally inconspicuous in the present group.

## Data Availability

The datasets used and/or analysed during the current study are available from the corresponding author on reasonable request.

## Abbreviations

ALADA: As Low As Diagnostically Acceptable
ALARA: As Low As Reasonably Achievable
ANB: A point–Nasion–B point angle
CBCT: Cone-beam computed tomography
DMS VI: Sixth German Oral Health Study
FF/RF: Forefoot/rearfoot load
FS: Frontal sway
FSW: Frontal sway width
IQR: Interquartile range
JMA: Jaw Motion Analyzer
L1–NB: Inclination of the mandibular central incisor relative to Nasion–B point line
L/R: Left/right
LCD: Liquid crystal display
PAS: Posterior airway space
S1–S3: Skeletal maturation stages according to the cervical vertebral maturation method
SNA: Sella–Nasion–A point angle
SNB: Sella–Nasion–B point angle
SS: Sagittal sway
U1–NA: Inclination of the maxillary central incisor relative to Nasion–A point line
